# Ciclopirox induces autophagy through reactive oxygen species-mediated activation of JNK signaling pathway

**DOI:** 10.18632/oncotarget.2471

**Published:** 2014-10-04

**Authors:** Hongyu Zhou, Tao Shen, Chaowei Shang, Yan Luo, Lei Liu, Juming Yan, Yan Li, Shile Huang

**Affiliations:** ^1^ State Key Laboratory of Phytochemistry and Plant Resources in West China, Kunming Institute of Botany, Chinese Academy of Sciences, Kunming 650201, China; ^2^ Department of Biochemistry and Molecular Biology, Louisiana State University Health Sciences Center, Shreveport, LA 71130-3932, USA; ^3^ Feist-Weiller Cancer Center, Louisiana State University Health Sciences Center, Shreveport, LA 71130-3932, USA

**Keywords:** Ciclopirox, autophagy, rhabdomyosarcoma, reactive oxygen species, JNK

## Abstract

Ciclopirox olamine (CPX), a fungicide, has been demonstrated as a potential anticancer agent. However, the underlying anticancer mechanism is not well understood. Here, we found that CPX induced autophagy in human rhabdomyosarcoma (Rh30 and RD) cells. It appeared that CPX-induced autophagy was attributed to induction of reactive oxygen species (ROS), as N-acetyl-L-cysteine (NAC), a ROS scavenger and antioxidant, prevented this process. Furthermore, we observed that CPX induced activation of mitogen-activated protein kinases (MAPKs), including extracellular signal-regulated kinase 1/2 (ERK1/2), c-Jun *N*-terminal kinase (JNK) and p38 MAPK, which was also blocked by NAC. However, only inhibition of JNK (with SP600125) or expression of dominant negative c-Jun partially prevented CPX-induced autophagy, indicating that ROS-mediated activation of JNK signaling pathway contributed to CPX-induced autophagy. Of interest, inhibition of autophagy by chloroquine (CQ) enhanced CPX-induced cell death, indicating that CPX-induced autophagy plays a pro-survival role in human rhabdomyosarcoma cells. Our finding suggests that the combination with autophagy inhibitors may be a novel strategy in potentiating the anticancer activity of CPX for treatment of rhabdomyosarcoma.

## INTRODUCTION

Ciclopirox olamine (CPX) (also called Batrafen, Loprox, Penlac and Stieprox), a synthetic hydroxypyridone derivative, is currently used for the treatment of superficial fungal infections and available in a variety of formulations, including cream, lotion, gel, nail lacquer and shampoos [1]. CPX has a broad spectrum of action against dermatophytes, yeast, filamentous fungi and bacteria. Mechanistically, CPX has been proposed to act as an iron chelator, forming complexes with trivalent metal cations, such as Fe^3+^, and inhibiting metal-dependent enzymes, such as catalase and peroxidase, which play an essential role in the intracellular degradation of toxic peroxides, though this remains to be determined [2]. Recent studies have shown that CPX induces cell death in different cancer cells, such as primary human acute myeloid leukemia cells, human breast cancer MDA-MB231 cells and human rhabdomyosarcoma Rh30 cells [3, 4]. However, the mechanism by which CPX induces cancer cell death is only at the beginning to be investigated. Eberhard *et al*. reported that CPX displays preclinical anticancer activity against hematologic malignancies and induces cell death through its ability to chelate intracellular iron and inhibit the iron-dependent enzyme ribonucleotide reductase [4]. We showed that CPX inhibits tumor growth in human breast cancer MDA-MB231 xenografts and induces cell death through caspase-dependent and caspase-independent mechanisms in Rh30 cells [3].

Autophagy is an evolutionarily conserved catabolic process that involves the degradation of the components of a cell through the lysosomal machinery [5]. During autophagy, a portion of the cytoplasmic materials and intracellular organelles are sequestered into a double membrane organelles known as autophagosomes, which degrade the sequestered contents by fusion with lysosomes to form autolysosomes [5]. Under some conditions, autophagy contributes to cellular survival by providing nutrients and energy to help cells adapt to starvation or stress (such as hypoxia, X-ray and anticancer drugs) [6]. However, under other settings, activated autophagy leads to cell death, called autophagic cell death or type II programmed cell death [6].

Increasing evidence has implicated that multiple stress conditions such as oxidative stress [7], endoplasmic reticulum (ER) stress [8] and pathogen infection [9], can induce autophagy through different molecular pathways. Among them, reactive oxygen species (ROS) function as signaling molecules not only in cell growth, differentiation, proliferation and apoptosis [10], but also in autophagy [11]. For example, hydrogen peroxide and 2-methoxyestradiol, two ROS-generating agents, can induce autophagic cell death in transformed HEK293 cells and cervical cancer (HeLa) cells [12]. Under starvation or oxidative stress conditions, ROS are increased and play a critical role in autophagosome formation through targeting cysteine protease Atg 4 [13]. Although the role of ROS in the regulation of autophagy has been confirmed, what signaling molecules involved in ROS-induced autophagy are still not well understood.

Mitogen-activated protein kinases (MAPKs) are evolutionarily conserved dual (Tyr and Ser/Thr) protein kinases, and play an important role in signal transduction from the cell surface to the nucleus [14]. To date, different groups of MAPKs have been characterized in mammals: the extracellular signal-regulated kinases ERK1/2, ERK3/4, ERK5, ERK7/8, the Jun N-terminal kinases JNK1/2/3 and the p38 MAPKs p38α/β/γ/δ [14]. Not only receptor-ligand interactions, but also different stress stimuli such as the oxidative stress caused by ROS, can induce potential activation of MAPK pathways [15]. Depending on the cell type and the stimulus, ERK signaling pathway mediates different cell responses, such as proliferation, apoptosis and autophagy [14–16]. Generally, growth factors may activate the RAS-RAF-MEK1/2-ERK1/2 pathway [14]. More recently, ERK5, also termed big mitogen-activated protein kinase-1 (BMK1), has been identified as a component of a parallel MAPK pathway, which is associated with a diverse range of cellular processes including cellular proliferation, migration, survival and angiogenesis [17]. The demonstration that the commonly used MEK1/2 inhibitors inactivated ERK5 suggested that ERK5 might regulate some of the cellular functions originally attributed to MEK1/2 [17, 18]. Studies have demonstrated that mammalian p38 MAPK has four isoforms: p38α, p38β, p38γ and p38δ, of which p38α is ubiquitously expressed, and best characterized [14,19]. p38 MAPK plays an essential role in the regulation of many cellular events including inflammation, cell growth, death, and differentiation [14, 19, 20]. Recently it has been found that p38 MAPK can also mediate autophagy in response to chemotherapeutic agents [21]. JNK, also known as the stress-activated protein kinase, has been implicated in apoptosis and autophagy [21–23]. In mammals, there are three JNK genes: JNK1, JNK2, and JNK3 [24]. JNK1 and JNK2 are ubiquitously expressed, while JNK3 is mainly expressed in brain, cardiac smooth muscle and testes [24]. In addition to apoptosis, JNK also contributes to autophagic induction in response to stress signals [21]. Particularly, ROS can induce JNK-dependent autophagy [25, 26].

Here we found that CPX induced autophagy in rhabdomyosarcoma (Rh30 and RD) cells, which was mediated by ROS induction, leading to activation of JNK cascade. Further, inhibition of autophagy by chloroquine (CQ) increased the cell death induced by CPX, indicating that CPX-induced autophagy played a pro-survival role in human rhabdomyosarcoma cells. Our findings suggest that combination treatment with CPX and pharmacological autophagy inhibitors might be a promising strategy for rhabdomyosarcoma therapy.

## RESULTS

### CPX reduces cell viability and alters morphology in rhabdomyosarcoma cells

**Figure 1:**
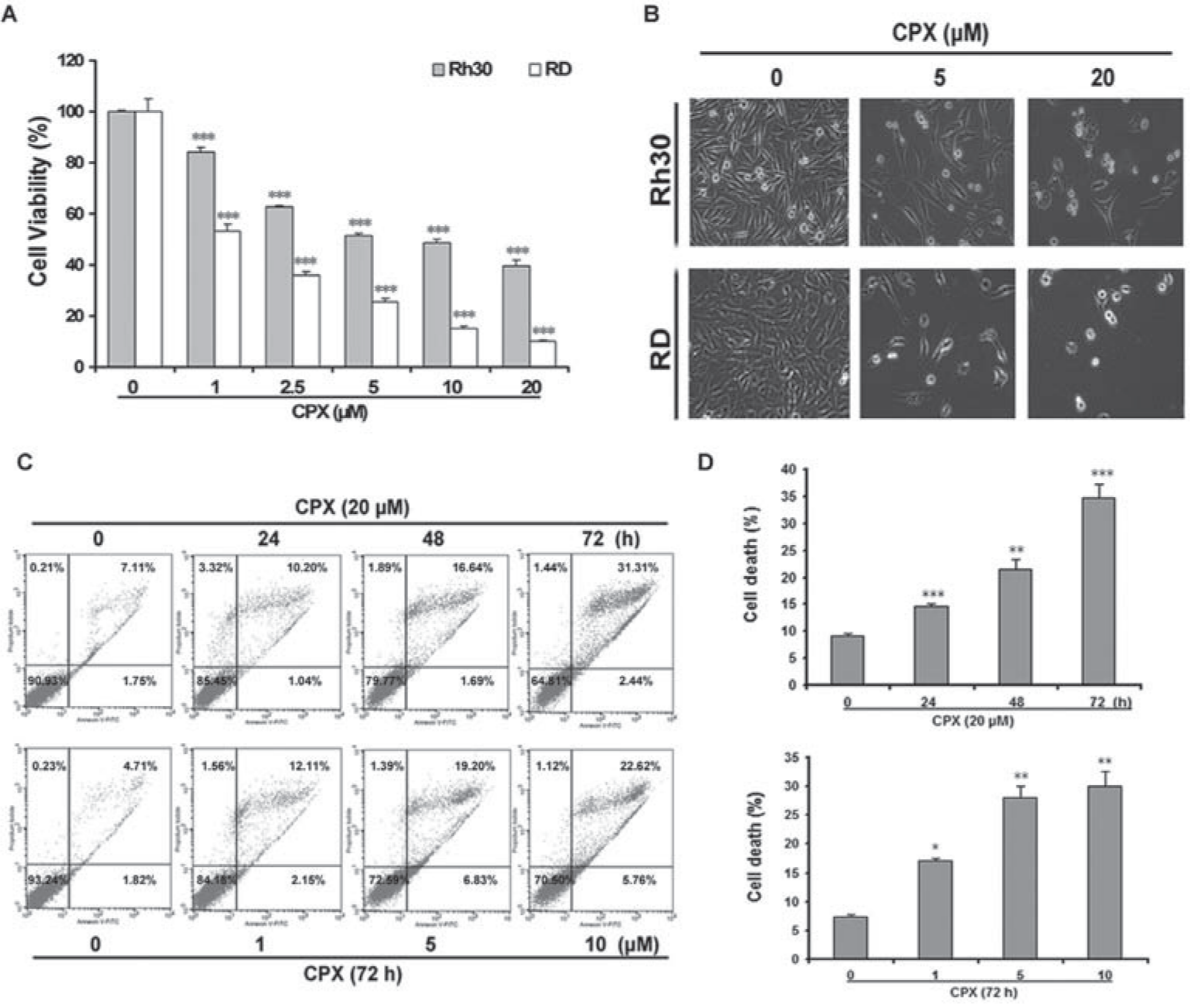
CPX decreases cell viability in rhabdomyosarcoma cells **(A)** RD and Rh30 cells were treated with indicated concentrations of CPX for 72 h. Cell viability was determined by MTS assay. **(B)** Rh30 and RD cells were treated with 5 and 20 μM of CPX for 72 h, followed by taking images under an Olympus inverted phase-contrast microscope equipped with the Quick Imaging system. **(C)** RD cells were treated with 0 and 20 μM of CPX for 0–72 h or incubated with 0–10 μM of CPX for 72 h. The cells were then harvested and processed for Annexin V-FITC/PI staining and flow cytometry. Results are presented as mean ± SD (n=3). *P < 0.05, **P < 0.01, ***P < 0.001, difference versus control group.

To investigate the cytotoxicity of CPX in rhabdomyosarcoma cells, RD and Rh30 cells were treated with various concentrations of CPX for 72 h, followed by MTS assay. As shown in Figure 1A, CPX decreased the cell viability in both RD and Rh30 cells in a concentration-dependent manner. By phase-contrast microscopic observation, more cells became round or detached from the culture plate when exposed to 20 μM of CPX (Figure 1B). To determine whether CPX causes cell death by inducing apoptosis, cells were analyzed by flow cytometry following Annexin V-FITC and propidium iodide (PI) staining. As shown in Figure 1C, 20 μM of CPX treatment for 48 and 72 h significantly increased the percentage of apoptotic cells compared with control cells.

### CPX induces autophagy in rhabdomyosarcoma cells

**Figure 2:**
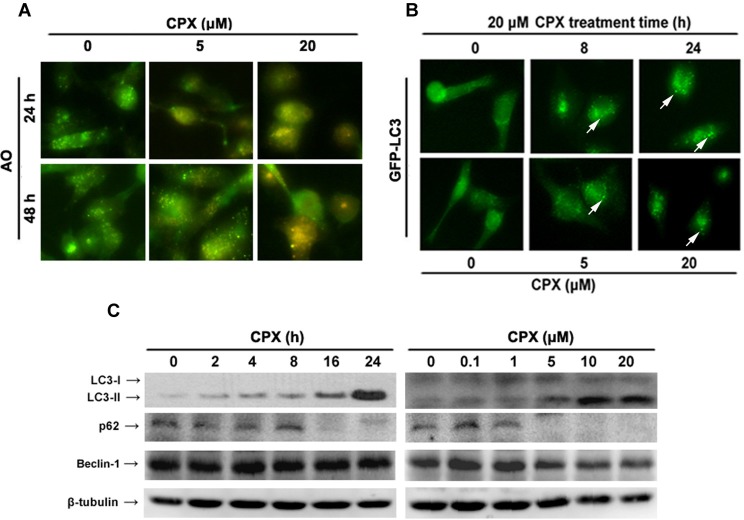
CPX induces autophagy in rhabdomyosarcoma cells **(A)** Representative images of AO staining of RD cells following treatment with 0–20 μM of CPX for 24 and 48 h. Red color intensity shows acidic vesicular organelles, representing autophagolysosomes. **(B)** Representative micrographs of cells that show GFP-LC3 localization. Rh30 cells stably expressing GFP-LC3 were treated without or with 20 μM of CPX for indicated time (Upper panel), or with indicated concentrations of CPX for 24 h (Bottom panel), and then visualized under a fluorescent microscope. Control cells presented a diffuse distribution of GFP-LC3, whereas CPX-treated cells displayed a punctate pattern of GFP-LC3 expression (arrows), indicating formation of autophagosomes. **(C)** Western blot analysis of autophagy-related proteins. RD cells were treated without or with 20 μM of CPX for indicated time, or with indicated concentrations of CPX for 24 h. The cells were harvested and subjected to Western blot analysis using indicated antibodies. β-tubulin was used as a loading control.

Recently we have demonstrated that CPX induces caspase-dependent and independent cell death in Rh30 cells [3]. Since autophagy contributes to cell death in some cases [6], here we studied whether CPX induces autophagy in the cancer cells. Autophagy is characterized by the increased acidic vesicular organelles (AVOs), which are correlated with increased autophagosomes. We therefore investigated whether CPX could induce autophagy by staining with acridine orange (AO) in RD cells. As shown in Figure 2A, 20 μM of CPX treatment for 24 h or 48 h induced the accumulation of AVO in the cytoplasm of RD cells.

LC3 is associated with the formation of the autophagosome membrane and is a specific marker for autophagy initiation [5]. Under normal conditions, LC3 is distributed homogeneously in the cytoplasm; when autophagy is induced, LC3 is recruited to the autophagosomal membrane and shows characteristic of GFP-LC3 puncta [5]. To elucidate whether CPX induced autophagy in rhabdomyosarcoma cells, the effect of CPX on the cellular localization of LC3 using Rh30 cells stably expressing GFP-LC3 was evaluated. As shown in Figure 2B, CPX treatment induced a punctuated fluorescent pattern of LC3, whereas untreated cells manifested a diffuse distribution of GFP-LC3, suggesting that CPX indeed induced autophagy in the cells.

Since LC3-I is converted to the hallmark autophagosome-associating protein LC3-II, expression of LC3-II has been widely used for monitoring autophagy [27]. To verify the above finding, we further tested whether CPX increases LC3-II protein level. The Western blotting results indicated that CPX potently increased LC3-II level in a time- and concentration-dependent manner in RD cells (Figure 2C). Moreover, induction of autophagy by CPX was identified by assessing the expressions of Beclin-1, a key regulator of autophagosome formation and p62/SQSTM1, a protein facilitating autophagic degradation of ubiquitinated protein aggregation [28]. CPX treatment decreased the expression of p62, but did not affect Beclin-1 expression (Figure 2C). Taken together, these results demonstrate that CPX induces autophagy in rhabdomyosarcoma cells.

### CPX induces autophagy via ROS induction

**Figure 3:**
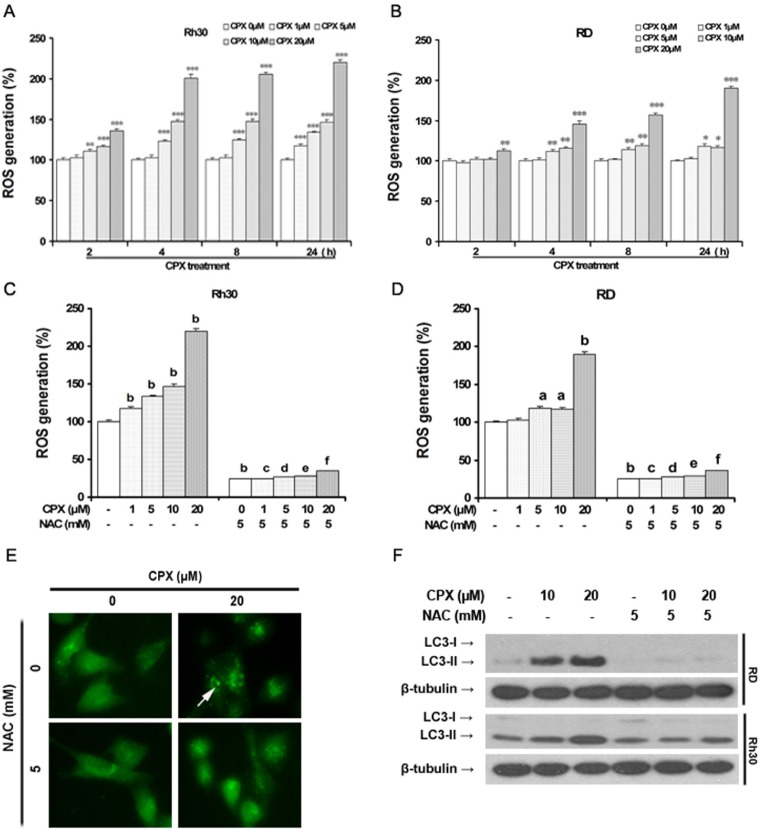
CPX increases intracellular level of ROS, thereby inducing autophagy **(A)** *and* **(B)** RD and Rh30 cells were treated without or with different concentrations of CPX (1–20 μM) for 30 min, followed by loading with 10 μM of CM-H_2_DCFDA for indicated time. Fluorescent intensity was detected using a microplate reader. Results are presented as mean ± SD (n=3). *P < 0.05, **P < 0.01, ***P < 0.001, difference versus control group. **(C)** *and* **(D)** RD and Rh30 cells were pre-incubated with or without NAC (5 mM) for 30 min, and then treated with or without various concentrations of CPX (0–20 μM) for 30 min, followed by loading with 10 μM of CM-H_2_DCFDA for 8 h. Fluorescent intensity was detected using a microplate reader. Results are presented as mean ± SD (n=3). ^a^*P* < 0.05, ^b^*P* < 0.01, difference versus control group. ^c^*P* < 0.001, difference versus 1 μM CPX group, ^d^*P* < 0.001, difference versus 5 μM CPX group, ^e^*P* < 0.001, difference versus 10 μM CPX group, ^f^*P* < 0.001, difference versus 20 μM CPX group. **(E)** Rh30 cells stably expressing GFP-LC3 were pretreated with 5 mM of NAC for 1 h, and then incubated with 0 and 20 μM of CPX for 24 h. The cells were visualized under a fluorescent microscope. **(F)** RD and Rh30 cells were pretreated with 5 mM of NAC for 1 h, and then incubated with 0–20 μM of CPX for 24 h. The cells were harvested and subjected to Western blot analysis with indicated antibodies. β-tubulin was used as a loading control.

Growing evidence shows that ROS are important regulators of autophagy under various conditions [11]. To determine whether CPX-induced autophagy is associated with ROS induction, we first measured ROS level in both RD and Rh30 cells treated with CPX using the ROS-detecting fluorescent dye CM-H_2_DCFDA. ROS accumulation was observed after CPX treatment with the culturing time, and increased ~2-fold after 24 h of CPX (20 μM) treatment in Rh30 cells (Figure 3A). Similar results were seen in RD cells (Figure 3B). Addition of 5 mM of NAC, a ROS scavenger, almost completely blocked CPX-induced ROS in both Rh30 and RD cells (Figure 3C and 3D). Interestingly, pretreatment with NAC remarkably attenuated CPX-induced GFP-LC3 puncta formation (Figure 3E) and LC3-II expression in the cells (Figure 3F), indicating that CPX-induced autophagy is mediated by ROS induction.

### ROS-mediated JNK activation contributes to CPX-induced autophagy

**Figure 4:**
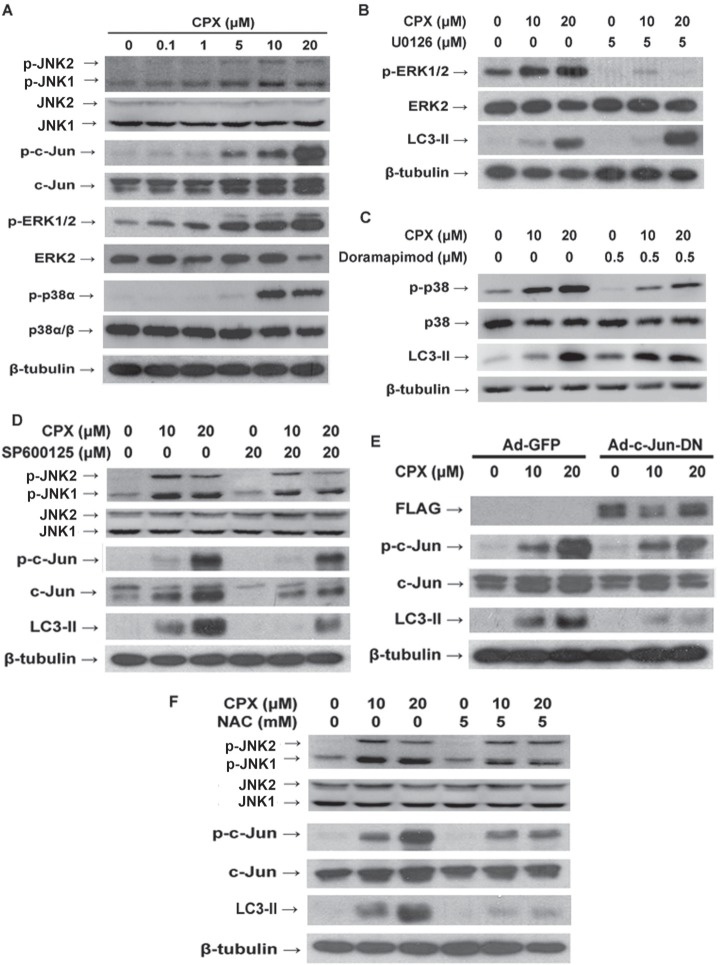
ROS-activated MAPKs pathway and JNK pathway contributes to CPX-induced autophagy **(A)** RD cells were incubated with different concentrations of CPX for 24 h, followed by Western blot analysis with indicated antibodies. **(B)** *and* **(C)** RD cells were pretreated with U0126 (5 μM) or doramapimod (0.5 μM) for 1 h, and then incubated with 0–20 μM of CPX for 24 h, followed by Western blot analysis with indicated antibodies. **(D)** RD cells were pretreated with SP600125 (20 μM) for 1 h, and then incubated with 0–20 μM of CPX for 24 h. The cells were harvested and subjected to Western blot analysis with indicated antibodies. **(E)** RD cells were infected with Ad-c-Jun-DN or Ad-GFP for 24 h, and then treated with CPX for 24 h. The cells were harvested and subjected to Western blot analysis with indicated antibodies. **(F)** NAC attenuated CPX-induced JNK activation and autophagy. RD cells were pretreated with NAC (5 mM) for 1 h, and then incubated with 0–20 μM of CPX for 24 h. The cells were harvested and subjected to Western blot analysis with indicated antibodies. β-tubulin was used as a loading control in A-F.

ROS have been demonstrated as an inducer or mediator for the activation of MAPK family members, including JNK, p38 and ERK1/2 [14]. Also, studies have shown that MAPKs play a pivotal role in autophagy [16, 21, 29]. In this study, we observed that CPX induced phosphorylation of p38α (Thr180/Tyr182), ERK1/2 and JNK1/2 (Thr183/Tyr185) in a concentration-dependent manner (Figure 4A). Thus, we next asked whether CPX-induced autophagy involves these MAPKs. To this end, selective inhibitors of ERK1/2, p38 and JNK were employed. As shown in Figure 4B, pretreatment with U0126, a highly selective inhibitor of MEK1/2 (upstream kinases of ERK1/2), or doramapimod (also named BIRB 796), a highly selective p38α MAPK inhibitor [30], markedly suppressed CPX-induced phosphorylation of ERK1/2 or p38, respectively. However, neither of the inhibitors apparently affected CPX-induced LC3-II expression, suggesting that ERK1/2 and p38 MAPK are not involved in CPX-induced autophagy. By contrast, presence of JNK inhibitor, SP600125, potently inhibited the activation of JNK pathway and LC3-II expression induced by CPX (Figure 4D). To further confirm the role of JNK signaling pathway in CPX-induced autophagy, RD cells were infected with recombinant adenovirus encoding FLAG-tagged dominant negative c-Jun (Ad-c-Jun-DN) or Ad-GFP (as control), and then treated with CPX for 24 h. As shown in Figure 4E, ectopic expression of dominant negative c-Jun substantially attenuated CPX-induced LC3-II, demonstrating that activation of JNK pathway contributes to CPX-induced autophagy.

To further investigate the role of ROS induction in CPX-induced autophagy and activation of JNK pathway, ROS scavenger NAC was utilized. We found that pre-treatment with NAC (5 mM) obviously attenuated CPX-induced c-Jun phosphorylation and LC3-II expression (Figure 4F), indicating that CPX induces autophagy through ROS-mediated activation of JNK signaling pathway.

### Autophagy plays a protective role in CPX-treated rhabdomyosarcoma cells

**Figure 5:**
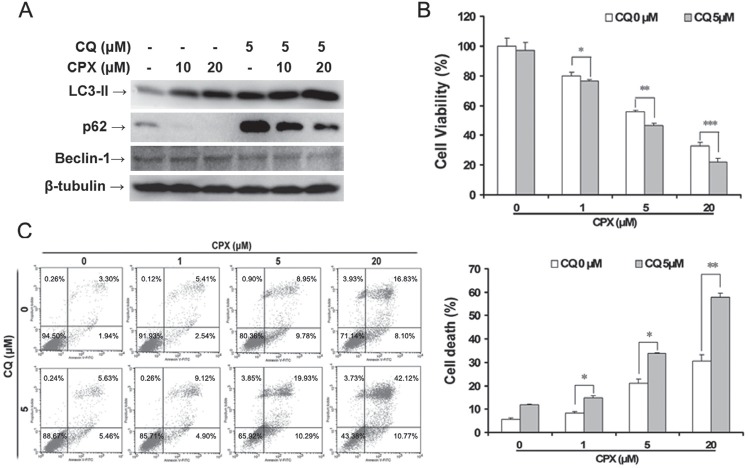
Inhibition of autophagy by CQ enhances CPX-induced autophagy and cell death **(A)** RD cells were pretreated with 5 μM of CQ for 1 h, and then incubated with 0–20 μM of CPX for 24 h. The cells were harvested and subjected to Western blot analysis with indicated antibodies. β-tubulin was used as a loading control. **(B)** RD cells were pretreated with 5 μM of CQ for 1 h, and then incubated with 0–20 μM of CPX for 48 h. Cell viability was measured by MTS assay. Results are presented as mean ± SD (n=3). *P < 0.05, difference versus 1 μM CPX group, **P < 0.01, difference versus 5 μM CPX group, ***P < 0.001, difference versus 20 μM CPX group. **(C)** RD cells were pretreated with 5 μM of CQ for 1 h, and then incubated with 0–20 μM of CPX for 48 h. The cells were then harvested and processed for Annexin V-FITC/PI staining and flow cytometry. Results are presented as mean ± SD (n=3). *P < 0.05, difference versus 1 μM and 5 μM CPX group, respectively, **P < 0.01, difference versus 20 μM CPX group.

Since autophagy has dual roles in cell survival and cell death [6, 31], to determine whether CPX-induced autophagy is pro-survival or pro-death in rhabdomyosarcoma cells, CQ, a pharmacologic autophagy inhibitor [5, 6], which inhibits the late autophagic process by blocking the fusion of autophagosomes and lysosomes, was used. As expected, treatment with CQ alone increased LC3-II and p62 expressions (Figure 5A). CPX-induced LC3-II expression and p62 degradation were elevated in the presence of 5 μM of CQ (Figure 5A). Importantly, MTS assay showed that inhibition of the late autophagic process with CQ (5 μM) for 48 h reduced the cell viability very marginally, but potentiated the CPX-reduced cell viability significantly (Figure 5B). Furthermore, Annexin V-PI staining also demonstrated that CQ enhanced CPX-induced apoptotic cell death (Figure 5C). These data suggest that autophagy protects rhabdomyosarcoma cells from CPX-induced cell death.

## DISCUSSION

CPX is an off-patent drug and currently used for the treatment of skin and nail fungal infection [1]. Recently, CPX has also been found to possess anticancer properties, by inhibiting cell proliferation, inducing cell death, as well as inhibiting angiogenesis and lymphangiogenesis [3, 4, 32, 33]. Oral administration of CPX at a dose of 20–25 mg/kg/day for 24–28 days inhibits the growth of xenografted leukemia (MDAY-D2, K562 and OCI-AML2) and primary human acute myeloid leukemia (AML) cells, as well as breast cancer (MDA-MB231) cells by 65–75% compared with vehicle control, but does not display evidence of weight loss or gross organ toxicity in mice [3, 4]. Furthermore, a recent phase I clinical trial has demonstrated that oral administration of CPX at a dose of 40 mg/m^2^ once daily for 5 days is well tolerated in all patients without dose-limiting toxicity, displays persistent pharmacodynamic activity in the patients, and induces disease stabilization and/or hematologic improvement in 2/3 patients with advanced hematologic malignancies [34]. Taken together, these findings highlight that CPX, as a fungicide, is a very promising anticancer agent and may be repurposed for cancer therapy.

Though CPX has been used as a fungicide for over 20 years, its antifungal mechanism is still not well understood. However, it has been proposed that CPX acts as an iron chelator, forming complexes with trivalent metal cations, such as Fe^3+^, and inhibiting iron-dependent enzymes, such as catalase and peroxidase, which catalyze degradation of toxic peroxides, resulting in oxidative toxicity in fungi [35]. Recently, it has been found that CPX induces cell death by chelating intracellular iron and inhibiting the iron-dependent enzyme ribonucleotide reductase in human leukemia and myeloma cells [4]. CPX induces apoptosis by increasing caspase-3/7 activity and down-regulating of protein expressions of Bcl-xL and survivin in human rhabdomyosarcoma cells [3]. Furthermore, CPX inhibits Wnt/β-catenin pathway [36], the mammalian target of rapamycin (mTOR) activity [37], and the eukaryotic translation initiation factor 5A (eIF5A) function [32, 38]. It is unknown whether iron chelation is implicated in these effects of CPX. Clearly, the antitumor mechanism of CPX is complex and needs to be further investigated.

In the present study, for the first time, we found that CPX induced autophagy in human rhabdomyosarcoma RD and Rh30 cells. Although the precise mechanism of autophagy remains unclear, growing evidence has implicated that ROS play a critical role in controlling autophagy. ROS are highly reactive molecules formed by the incomplete one-electron reduction of oxygen, including oxygen ions and peroxides [39]. ROS form as a natural byproduct of the normal metabolism of oxygen and participate in cell signaling and homeostasis at low levels. However, under environmental stress, high levels of ROS can cause irreversible oxidative damage to cell structures [39]. Several stimuli that induce ROS generation can also induce autophagy, such as nutrient starvation, hypoxia, oxidative stress and some chemotherapeutic agents [7]. Alterations in ROS levels and autophagy play a crucial role in cancer initiation and progression, and both are recognized as the potential targets for cancer treatment [31, 40]. Our present data showed that CPX induced ROS, which were detectable in 4 h and increased by approximately 2-fold in 24 h of treatment. NAC, the ROS scavenger, remarkably attenuated CPX-induced GFP-LC3 puncta formation and LC3-II expression. Our results suggest that CPX triggers autophagy by induction of ROS.

Multiple signaling molecules, such as MAPKs, mTOR, and class III PI3K, have been shown to regulate autophagy [24, 41]. The MAPKs, including ERK1/2, JNK and p38 MAPK, are a family of serine/threonine kinases that regulate a variety of cellular events such as proliferation and apoptosis [42]. It is well known that activation of JNK contributes to stress-induced apoptosis [42]. Recent studies have further revealed that activation of JNK is also associated with autophagy induction [24]. Endoplasmic reticulum (ER) stress induces autophagosome formation and accumulation by activation of inositol-requiring enzyme 1 (IRE1)-JNK pathway [43]. Bufalin induces autophagy via ROS induction and JNK activation [44]. Here we found that CPX was able to activate p38α, ERK1/2, and JNK1/2, but only activation of JNK pathway was responsible for CPX-induced autophagy. This is strongly supported by the findings that inhibition of JNK with SP600125 or ectopic expression of dominant negative c-Jun potently inhibited CPX-induced autophagy, whereas inhibition of ERK1/2 and p38α using U0126 and doramapimod, respectively, failed to prevent CPX-induced autophagy. These results suggest that JNK pathway plays a critical role in CPX-induced autophagy. Our further study showed that pre-treatment with NAC prevented CPX-induced phosphorylation of c-Jun and autophagy, indicating that CPX-induced autophagy is dependent on ROS-activated JNK cascade. Further research is needed to address whether CPX induces autophagy also by targeting mTOR and class III PI3K.

Recent studies have shown that several anticancer agents including tamoxifen, 5-fluorouraci and rapamycin can induce autophagy [45–47]. However, the role of autophagy in cancer therapy is complex and controversial. It has been reported that increased autophagy may be pro-apoptotic [48] or pro-survival [45], depending on experimental conditions (cell types and anticancer agents). Some efforts have been made to find under what conditions autophagy can be exploited for cancer therapy. Of note, inhibition of autophagy enhances vorinostat or tamoxifen-induced apoptosis [46, 49]. Here we observed that disruption of autophagy using CQ increased CPX-induced apoptosis, indicating that CPX-induced autophagy is a pro-survival mechanism in rhabdomyosarcoma cells.

In conclusion, we show that CPX induced autophagy in human rhabdomyosarcoma cells. CPX-induced autophagy was mediated by ROS induction and JNK activation. Disruption of autophagy using CQ enhanced CPX-induced cell death, indicating that CPX-induced autophagy is a pro-survival mechanism in the cells. Our findings suggest that combination of CPX and an autophagy inhibitor (e.g. CQ) may be a promising strategy for cancer therapy.

## MATERIAL AND METHODS

### Cell culture, agents and antibodies

Human rhabdomyosarcoma (Rh30 and RD) cell lines (gifts from Peter J. Houghton, Nationwide Children's Hospital, Columbus, OH) were grown in antibiotic-free RPMI 1640 (Mediatech, Herndon, VA) supplemented with 10% fetal bovine serum (FBS) (Atlanta Biologicals, Lawrenceville, GA). Rh30 cells stably expressing green fluorescent protein-light chain 3 (GFP-LC3) were generated by transfection of Rh30 cells with pEGFP-LC3 plasmid (Addgene, Cambridge MA) using Lipofectamine 2000 (Life Technologies, Grand Island, NY) and selection with G418 (500 μg/ml) (Mediatech). Cells were maintained in a humid incubator (37°C, 5% CO_2_). Ciclopirox olamine (CPX) (Sigma, St. Louis, MO) was dissolved in 100% ethanol to prepare a stock solution (100 mM), then aliquoted and stored at −20°C. Enhanced chemiluminescence solution was from Perkin-Elmer Life Science (Boston, MA, USA). CellTiter 96^®^ A_Queous_ One Solution Cell Proliferation Assay kit was from Promega (Madison, WI). Annexin V-FITC Apoptosis Detection Kit I was purchased from BD Biosciences (San Jose, CA). U0126, SP600125 and doramapimod were obtained form LC Laboratories (Woburn, MA). N-acetylcysteine (NAC), AO and CQ were purchased from Sigma. 5–6-chloromethyl-2,7-dichlorodihydrofluorescein diacetate (CM-H_2_DCFDA) was from Life Technologies. The following antibodies were used: p-JNK (Thr183/Tyr185), JNK, p-c-Jun (Ser63), c-Jun, ERK2, p-p38 (Thr180/Tyr182), p38α/β, p62, Beclin-1, MAP LC3β (Santa Cruz Biotechnology, Santa Cruz, CA), phospho-p44/42 MAPK (ERK1/2) (Thr202/Tyr204) (Cell Signaling, Beverly, MA), β-tubulin, FLAG (Sigma), goat anti-rabbit IgG-horseradish peroxidase (HRP), goat anti-mouse IgG-HRP, and rabbit anti-goat IgG-HRP (Pierce, Rockford, IL).

### Cell viability assay

Cell viability was determined by MTS (3-(4, 5-dimethylthiazol-2-yl)-5-(3-carboxymethoxyphenyl)-2-(4-sulfophenyl)-2H-tetrazolium, inner salt) assay, according to the protocol of CellTiter 96^®^ A_Queous_ One Solution Cell Proliferation Assay kit (Promega). Briefly, 100 μl of cell suspensions were seeded into each well of 96-well plates (1×10^4^ cells/well) and incubate overnight. The cells were then exposed to the tested compounds in triplicates for 72 h, followed by adding 20 μl of One Solution Reagent per well. After further incubation at 37°C for 1–2 h, cell viability was determined by measuring the optical density (OD) at 490 nm using a Wallac 1420 Multilabel Counter (PerkinElmer Life Sciences, Wellesley, MA).

### Cell morphological analysis

Rh30 and RD cells were seeded in 6-well plates (2 × 10^5^ cells/well). The next day, the cells were treated with CPX (0, 5 and 20 μM). After incubation for 72 h, images were taken with an Olympus inverted phase-contrast microscope (Olympus Optical Co., Melville, NY) (200×) equipped with the Quick Imaging system.

### Apoptosis assay

Cells were pre-incubated with or without 5 μM of CQ for 1 h, and then treated with or without CPX (1, 5 and 20 μM) for 48 h. Cells were then collected and stained using Annexin V-FITC Apoptosis Detection Kit I (BD Biosciences) according to the manufacturer's instruction. In brief, cells were washed with cold PBS, and then resuspended in 100 μl of Annexin-V binding buffer, followed by incubation with FITC conjugated Annexin V and propidium iodide (PI) for 15 min at room temperature in the dark. Flow cytometry was performed using a FACS Calibur flow cytometer (Becton Dickinson, San Jose, CA). Cells treated with vehicle alone (100% ethanol) were used as a control.

### ROS detection

Intracellular level of ROS was measured by detecting the fluorescent intensity of oxidant-sensitive probe CM-H_2_DCFDA, which is taken up by cells, cleaved by esterases to DCFH and trapped intracellularly. Briefly, Rh30 and RD cells were seeded at a density of 1 × 10^4^ cells/well in 96-well plates. The next day, the cells were pre-incubated with or without NAC (5 mM) for 30 min, and then treated with or without CPX (0–20 μM) for 30 min, followed by loading with 10 μM of CM-H_2_DCFDA. At different time points (2, 4, 8 and 24 h), the fluorescent intensity was detected by excitation at 485 nm and emission at 535 nm using a Wallac 1420 Multilabel Counter (Perkin-Elmer Life Sciences, Wellesley, MA).

### GFP-LC3 puncta assay

Rh30 cells stably expressing GFP-LC3 were seeded at a density of 2 × 10^5^ cells/well in 6-well plates. The next day, the cells were treated without or with 20 μM of CPX for different time (8 and 24 h), or with different concentrations of CPX (0–20 μM) for 24 h, and examined under a fluorescence microscope.

### Western blot analysis

Cells were seeded in 6-well plates at a density of 5 × 10^5^ cells/well. The next day, the cells were treated with CPX (0–20 μM) for 24 h, or with 20 μM CPX for 0–24 h. For experiments with NAC or MAPK inhibitors, cells were pre-incubated with or without NAC or MAPK inhibitors for 1 h, respectively. The cells were then treated with or without CPX (10 and 20 μM) for 24 h. Cell lysis and immunoblotting were performed as described previously [3]. β-tubulin served as a loading control.

### Infection of cells with recombinant adenovirus

Recombinant adenoviruses expressing FLAG-tagged dominant negative c-Jun (FLAG-Δ169) (Ad-c-Jun-DN) and green fluorescence protein (GFP) (Ad-GFP) were described [50]. For experiments, RD cells were seeded in 6-well plates at a density of 5 × 10^5^ cells/well. The next day, the cells were infected with the Ad-c-Jun-DN or Ad-GFP (as a control) for 24 h at 5 of multiplicity of infection (MOI = 5). The cells were then treated with CPX (0–20 μM) for 24 h, followed by cell viability, morphology, and Western blot analysis. Expression of FLAG-tagged dominant negative c-Jun was confirmed by Western blotting with antibody to FLAG.

### Statistical analysis

Results were expressed as mean values ± standard deviation (mean ± SD). The data were analyzed by one-way analysis of variance (ANOVA) followed by post-hoc Dunnett's *t*-test for multiple comparisons. A level of *P* < 0.05 was considered to be statistically significant.
